# Reconsidering the T category for the T3 non-small cell lung cancer with additional tumor nodules in the same lobe: A population-based study

**DOI:** 10.3389/fonc.2023.1043386

**Published:** 2023-04-05

**Authors:** Jing-Sheng Cai, Fan Yang, Xun Wang

**Affiliations:** ^1^Department of Thoracic Surgery, Peking University People’s Hospital, Beijing, China; ^2^Thoracic Oncology Institute, Peking University People’s Hospital, Beijing, China

**Keywords:** non-small cell lung cancer, T3-Add, T3 category, T2b category, survival

## Abstract

**Background:**

This study aimed to evaluate the prognosis of the T3 non-small cell lung cancer (NSCLC) patients with additional tumor nodules in the same lobe (T3-Add), and externally validate the current T category of this population.

**Methods:**

NSCLC data deposited in the Surveillance, Epidemiology, and End Results (SEER) dataset was extracted. Survivals were estimated using the Kaplan-Meier method with a log-rank test. Propensity score matching (PSM) was performed to reduce bias. The least absolute shrinkage and selection operator (LASSO)-penalized Cox model was used to determine the prognostic factors.

**Results:**

A total of 41,370 eligible cases were included. There were 2,312, 20,632, 12,787, 3,374 and 2,265 cases in the T3-Add, T1, T2, T3 and T4 group, respectively. The Kaplan-Meier curves demonstrated that the survivals of the T3-Add patients were superior to those of the T3 patients both before and after PSM. Additionally, the OS of the T3-Add patients were worse than that of the T2 patients, but the CSS differences between these two groups were not statistically significant. In the subset analyses, the survivals of the T3-Add patients were inferior to those of the T2a patients, but were comparable to those of the T2b patients (5-year OS rate: 54.3% vs. 57.2%, *P* = 0.884; 5-year CSS rate: 76.2% vs. 76.8%, *P* = 0.370). In the T3-Add & T2b matched pair, multivariable Cox analysis further confirmed that T category was not a prognostic factor for survivals.

**Conclusion:**

T3-Add and T2b NSCLC patients had similar survivals, and we proposed that it is necessary to reconsider the T category of the patients with additional nodules in the same lobe in the forthcoming 9^th^ edition of TNM staging manual.

## Introduction

Lung cancer ranks first as the cause of cancer-related mortality worldwide (1, 2). Non-small cell lung cancer (NSCLC) accounts for about 85% of all the lung malignancies (3). The 8^th^ edition of the International Association for the Study of Lung Cancer (IASLC) proposed tumor-node-metastasis (TNM) staging manual for NSCLC (4) is considered as the predominating prognostic factor for NSCLC patients’ survivals, which guides treatment strategy selection. In recent years, much effort has been devoted to refine this staging manual to achieve a more accurate staging (5–8).

In the current TNM staging manual, tumors with additional nodules in the same lobe are assigned to T3 category (T3-Add) (4, 9). However, survival dispute arises over the T category of these patients (8), and more externally validations are needed. The previous report demonstrated that the overall survival (OS) of the T3-Add patients was superior to that of the remaining T3 patients, but was similar to that of T2b patients (8). Therefore, the authors proposed to reconsider these patients to T2b category (8). Given the great significance of the TNM staging manual, it is imperative to determine the proper T category of this population subset.

Against this background, the current study analyzed the resected NSCLC data recorded in the Surveillance, Epidemiology, and End Results (SEER) dataset and aimed to revealed the heterogeneity of prognosis between T3-Add and other patients. We hoped to answer the question that whether T3-Add patients should remain classified as T3 category.

## Methods

### Study population

**Figure 1:**
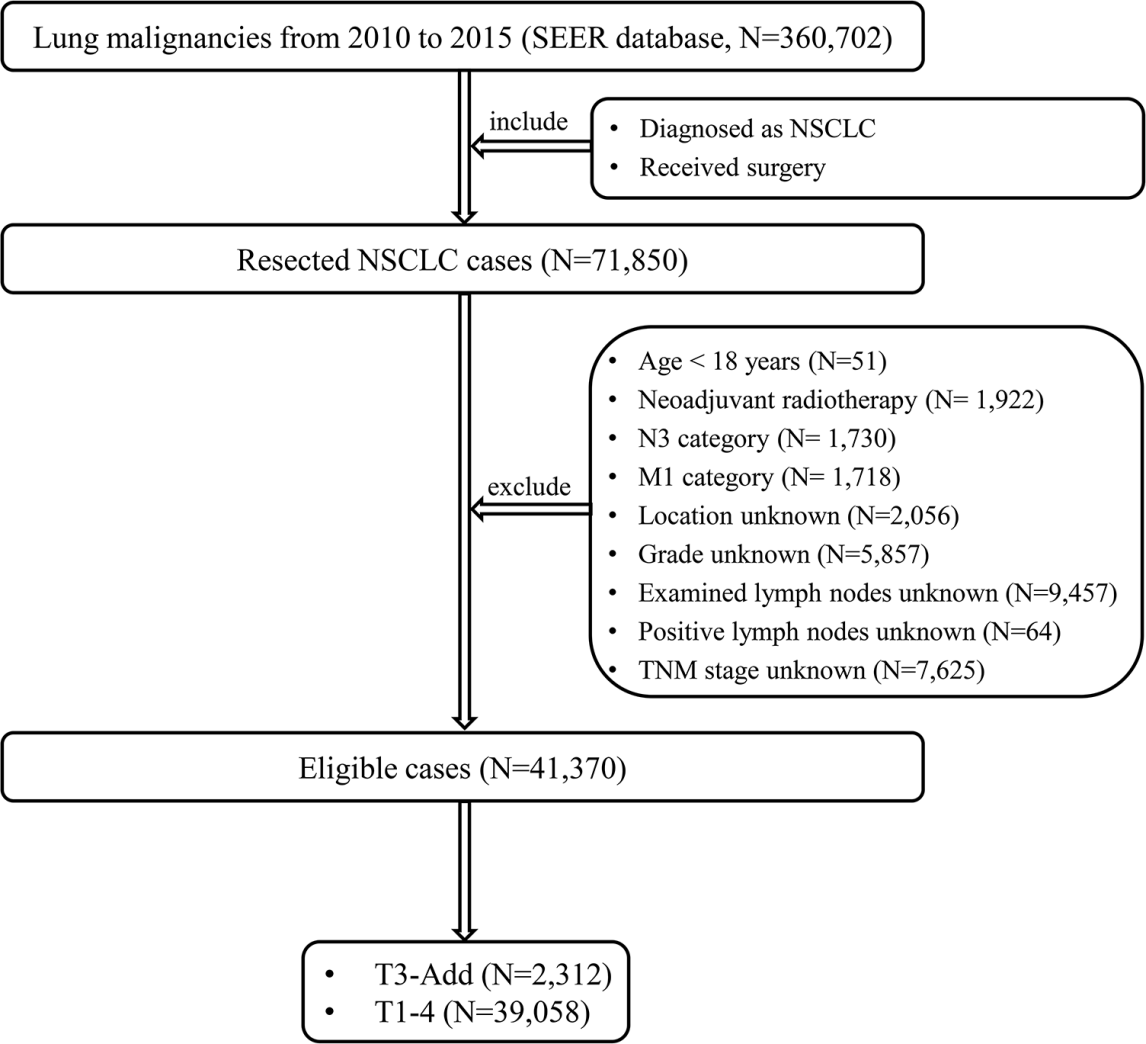
The patient selection flow chart. NSCLC, non-small cell lung cancer; TNM, tumor-node-metastasis; T3-Add, T3 non-small cell lung cancer with additional tumor nodules in the same lobe.

From 2010 to 2015, a total of 360,702 lung malignancies cases were extracted from the SEER database. The patient selection flow chart is showed in **Figure 1**. The eligible cases satisfy the following criteria: [1] diagnosed as NSCLC and [2] received surgery. The exclusion criteria mandated that: [1] age < 18 years; [2] neoadjuvant radiotherapy; [3] N3 category; [4] M1 category; [5] location unknown; [6] grade unknown; [7] examined lymph nodes unknown; [8] positive lymph nodes unknown and [9] TNM stage unknown. According to the CS Site-Specific Factor 1 code, T3-Add patients were selected (code 010: separate tumor nodules in ipsilateral lung, same lobe). At last, the included cases were assigned to five groups: T3-Add, T1, T2, T3 and T4 groups. This study mainly focused on the T3-Add, T2 and T3 patients.

### Ethic

This study was approved by the Ethics Committee of Peking University People’s Hospital. Permission was obtained to retrieve SEER data files using the reference number: 12962-Nov2019. Given the anonymous patient data and retrospective design, this study was dispensed with the informed consent forms.

### Data collection

The variables, including age, sex (male/female), tumor location (upper lobe/middle lobe/lower lobe), histology (adenocarcinoma/squamous cell carcinoma/other), grade (well/moderate/poor or undifferentiated), surgery (lobectomy/pneumonectomy/sublobectomy), radiotherapy (no and yes), chemotherapy (no and yes), examined lymph nodes, positive lymph nodes, tumor size, pathologic T category, pathologic N category, pathologic TNM stage, visceral pleural invasion (VPI), patient status, cause of death and survival time. The pathologic 8^th^ edition of the TNM staging manual (4) was used in this study. A complete data analysis was conducted in this study.

### Endpoints

Patients with exact survival time and definitive status were included. The primary endpoints of this study were OS and Cancer specific survival (CSS). OS was defined as the time period from the date of diagnosis to the date of death from any cause or the last follow-up. CSS was defined as the time period from the date of diagnosis to the date of death caused by NSCLC or the last follow-up. The median follow-up time was 33.0 months (range: 0.48-83.04 months).

### Statistical analysis

SEER*Stat software version 8.3.4. was used to extract the NSCLC data. R version 4.1.1 (The R Foundation for Statistical Computing, Vienna, Austria; http://www.r-project.org) and IBM SPSS Statistics (version 25.0, IBM Corp, Armonk, NY, USA) were used to conduct statistical analyses. Categorical variables were presented as numbers and percentages, which were compared using the Pearson χ2 test or Fisher’s exact test. Nonnormally distributed continuous variables were presented as median and range, which were compared using the Mann-Whitney U test. Survivals were analyzed using the Kaplan-Meier method with a log-rank test. A one to one propensity score matching (PSM) method was used to reduce the bias caused by the confounding variables in baseline characteristics using the R package “MatchIt” (10). The variables, including age, sex, histology, grade, surgery, radiotherapy, chemotherapy, N category and VPI, were included in the PSM models. The caliper distance of the T3-Add & T2 pair, T3-Add & T3 pair, T3-Add & T2a pair and T3-Add & T2b pair were 0.0001, 0.00001, 0.0001 and 0.00001, respectively. Least absolute shrinkage and selection operator (LASSO) regression model was used to minimize and select the potential prognostic factors using the R package “glmnet” (11). The variables, including age, sex, location, histology, grade, surgery, radiotherapy, chemotherapy, examined lymph nodes, positive lymph nodes, T category, N category, TNM stage and VPI, were included in the LASSO model. The LASSO-selected prognostic factors were further entered into a forward stepwise multivariable Cox model to determine the final independent prognostic factors. Two-sided P values < 0.05 was considered statistically significant.

## Results

### Patient characteristics

The aforementioned inclusion and exclusion criteria yielded a study population of 41,370 eligible cases. The baseline characteristics of the included patients are listed in **Table 1**. The median age was 69 years (range: 18-99 years). Over half of cases were female (52.8%). There were 2,312 cases, 20,632 cases, 12,787 cases, 3,374 cases and 2,265 cases in the T3-Add, T1, T2, T3 and T4 group, respectively. The incidence of the T3-Add patients was 5.6% (2,312/41,370). The 5-year OS rate of the T3-Add patients was 51.5%, and the 5-year CSS rate was 72.5%. When compared with the T2 group, more patients in the T3-Add group were female (*P* < 0.001), diagnosed with adenocarcinoma (*P* < 0.001), diagnosed with well differentiated diseases (*P* < 0.001), received radiotherapy (*P* < 0.001) and received chemotherapy (*P* < 0.001). In addition, less patients in the T3-Add group were suffered from VPI (*P* < 0.001). When compared with the T3 group, there were higher percentage of female (*P* < 0.001), upper lobe tumors (*P* < 0.001), adenocarcinoma (*P* < 0.001) and well differentiated diseases (*P* < 0.001) in the T3-Add group. More small-sized and early N category tumors were diagnosed in the T3-Add group (tumor size: *P* < 0.001; N category: *P* < 0.001). Less patients in the T3-Add group received pneumonectomy (*P* < 0.001), received radiotherapy (*P* = 0.001) and received chemotherapy (*P* < 0.001). After PSM, there were 1,623 cases, 650 cases, 1,381 cases and 788 cases in the T3-Add & T2 pair, T3-Add & T3 pair, T3-Add & T2a pair and T3-Add & T2b pair, respectively. The baseline characteristics were all balanced well after PSM (**Tables S1**, **S2**).

**Table 1:** The baseline characteristics of the included NSCLC patients.

Variables	Total (N= 41,370)	T3-Add (N=2,312)	T2 (N=12,787)	T3 (N=3,374)	*P1*^a^	*P2*^b^
Age, years					0.596^c^	0.921^c^
Median (range)	69 (18-99)	69 (24-91)	69 (19-96)	69 (18-95)		
Sex					< 0.001	< 0.001
Male	19,523 (47.2)	1,018 (44.0)	6,300 (49.3)	1,983 (58.8)		
Female	21,847 (52.8)	1,294 (56.0)	6,487 (50.7)	1,391 (41.2)		
Location					0.191	< 0.001
Upper lobe	24,586 (59.4)	1,411 (61.0)	7,643 (59.8)	1,919 (56.9)		
Middle lobe	2,448 (5.9)	114 (4.9)	744 (4.8)	132 (3.9)		
Lower lobe	14,336 (34.7)	787 (34.0)	4,400 (34.4)	1,323 (39.2)		
Histology					< 0.001	< 0.001
Adenocarcinoma	21,201 (51.2)	1,296 (56.1)	6,356 (49.7)	1,214 (36.0)		
Squamous cell carcinoma	10,264 (24.8)	396 (17.1)	3,455 (27.0)	1,449 (42.9)		
Other	9,905 (23.9)	620 (26.8)	2,976 (23.3)	711 (21.1)		
Grade					< 0.001	< 0.001
Well	8,028 (19.4)	485 (21.0)	1,632 (12.8)	259 (7.7)		
Moderate	18,906 (45.7)	1,032 (44.6)	6,031 (47.2)	1,260 (37.3)		
Poor/undifferentiated	14,436 (34.9)	795 (34.4)	5,124 (40.1)	1,855 (55.0)		
Surgery					0.027	< 0.001
Lobectomy	34,047 (82.3)	1,956 (84.6)	11,032 (86.3)	2,946 (87.3)		
Pneumonectomy	1,362 (3.3)	74 (3.2)	433 (3.4)	289 (8.6)		
Sublobectomy	5,961 (14.4)	282 (12.2)	1,322 (10.3)	139 (4.1)		
Radiotherapy					< 0.001	0.001
No	38,155 (92.2)	2,044 (88.4)	11,607 (90.8)	2,876 (85.2)		
Yes	3,215 (7.8)	268 (11.6)	1,180 (9.2)	498 (14.8)		
Chemotherapy					< 0.001	< 0.001
No	31,933 (77.2)	1,461 (63.2)	9,187 (71.8)	1,776 (52.6)		
Yes	9,437 (22.8)	851 (36.8)	3,600 (28.8)	1,598 (47.7)		
Examined lymph nodes					0.139^c^	< 0.001^c^
Median (range)	8 (1-90)	8 (1-90)	9 (1-82)	10 (1-90)		
Positive lymph nodes					0.044^c^	< 0.001^c^
Median (range)	0 (0-61)	0 (0-24)	0 (0-38)	0 (0-18)		
Tumor size, mm					< 0.001^c^	< 0.001^c^
Median (range)	25 (1-989)	25 (1-185)	35 (1-50)	58 (6-70)		
T category						
1	20,632 (49.9)					
2	12,787 (30.9)					
3	5,686 (13.7)					
4	2,265 (5.5)					
N category					0.090	< 0.001
0	3,2585 (78.8)	1,658 (71.7)	9,364 (73.2)	2,211 (65.5)		
1	5,087 (12.30)	350 (15.1)	1,945 (15.2)	722 (21.4)		
2	3,698 (8.9)	304 (13.1)	1,478 (11.6)	441 (13.1)		
TNM stage						
IA	17,946 (43.4)					
IB	7,382 (17.8)					
IIA	1,982 (4.8)					
IIB	7,371 (17.8)					
IIIA	5,598 (13.5)					
IIIB	1,091 (2.6)					
VPI					< 0.001	< 0.001
Without	32,479 (78.5)	1,720 (74.4)	6,576 (51.4)	2,068 (61.3)		
With	8,891 (21.5)	592 (25.6)	6,211 (48.6)	1,306 (38.7)		

a T3-Add vs. T2.

b T3-Add vs. T3.

c Mann–Whitney U test.

NSCLC, non-small cell lung cancer; T, tumor; T3-Add, T3 tumors with additional nodules in the same lobe; N, node; TNM, tumor-node-metastasis; VPI, visceral pleural invasion.

### Survival analysis

**Figure 2:**
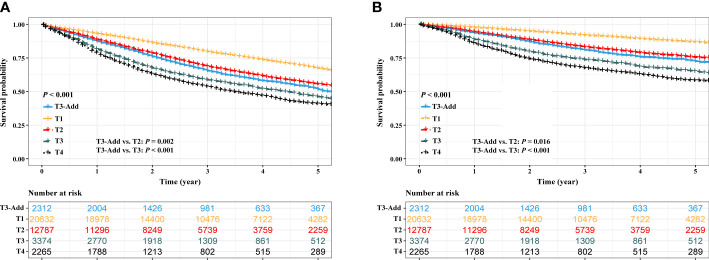
Kaplan–Meier estimates of survivals in the full analysis set. **(A)** overall survival: T3-Add vs. T1 vs. T2 vs. T3 vs. T4 and **(B)** cancer specific survival: T3-Add vs. T1 vs. T2 vs. T3 vs. T4. T3-Add, T3 non-small cell lung cancer with additional tumor nodules in the same lobe.

Before PSM, a progressively reduced OS and CSS were observed depending on T category (OS: *P* < 0.001, **Figure 2A**; CSS: *P* < 0.001, **Figure 2B**). The OS of the T3-Add patients was better than that of the T3 patients (5-year OS rate: 51.5% vs. 46.1%, *P* < 0.001), but was inferior to that of T2 patients (5-year OS rate: 51.5% vs. 55.6%, *P* = 0.002). Similar results were also observed in the CSS comparisons (5-year CSS rate: T3-Add vs. T3 = 72.5% vs. 55.6%, *P* < 0.001; 5-year CSS rate: T3-Add vs. T2 = 72.5% vs. 75.6%, *P* = 0.016).

**Figure 3:**
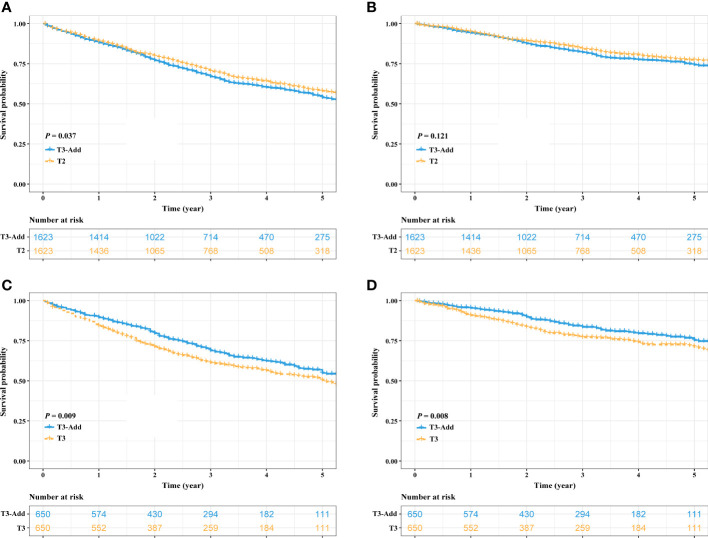
Kaplan–Meier estimates of survivals in the T3-Add & T2 pair and the T3-Add & T3 pair after PSM. **(A)** overall survival: T3-Add vs. T2; **(B)** cancer specific survival: T3-Add vs. T2; **(C)** overall survival: T3-Add vs. T3; **(D)** cancer specific survival: T3-Add vs. T3. PSM, propensity score matching; T3-Add, T3 non-small cell lung cancer with additional tumor nodules in the same lobe.

**Figure 4:**
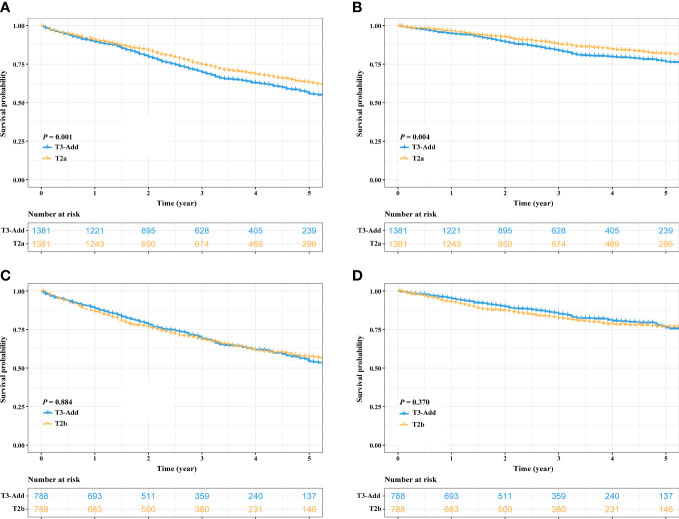
Kaplan–Meier estimates of survivals in the T3-Add & T2a pair and the T3-Add & T2b pair after PSM. **(A)** overall survival: T3-Add vs. T2a; **(B)** cancer specific survival: T3-Add vs. T2a; **(C)** overall survival: T3-Add vs. T2b; **(D)** cancer specific survival: T3-Add vs. T2b. PSM, propensity score matching; T3-Add, T3 non-small cell lung cancer with additional tumor nodules in the same lobe.

After PSM, considering the T3-Add & T2 matched pair, the OS of the T3-Add patients was still worse than that of the T2 patients (5-year OS rate: 53.9% vs. 58.0%, *P* = 0.037, **Figure 3A**). However, these two groups of patients had similar CSS (5-year CSS rate: 74.4% vs. 77.1%, *P* = 0.121, **Figure 3B**). Regarding the T3-Add & T3 matched pair, the survivals of the T3-Add patients were superior to those of the T3 patients (5-year OS rate: 54.8% vs. 50.4%, *P* = 0.009, **Figure 3C**; 5-year CSS rate: 75.3% vs. 71.2%, *P* = 0.008, **Figure 3D**). In the subset analyses, considering the T3-Add & T2a matched pair, T2a patients had longer survivals than T3-Add patients (5-year OS rate: 55.7% vs. 63.1%, *P* = 0.001, **Figure 4A**; 5-year CSS rate: 76.5% vs. 81.8%, *P* = 0.004, **Figure 4B**). Regarding the T3-Add & T2b matched pair, the survivals of the T3-Add patients were comparable to those of the T2b patients (5-year OS rate: 54.3% vs. 57.2%, *P* = 0.884, **Figure 4C**; 5-year CSS rate: 76.2% vs. 76.8%, *P* = 0.370, **Figure 4D**).

When stratified T3-Add patients based on tumor size, the data showed that T3-Add (0-30 mm) had the best survival rates (Both OS and CSS) than the remaining T3 patients (**Figure S1**, all *P* < 0.001). The OS of T3-Add (30-50 mm) was comparable to that of T3-Add (50-70 mm) patients (*P* = 0.474, **Figure S1A**), but was marginally better than that of T3-Add (> 70 mm) (*P* = 0.047, **Figure S1A**). T3-Add (50-70 mm) and T3-Add (> 70 mm) patients had similar OS (*P* = 0.252, **Figure S1A**). Considering CSS, diminishing CSS with increasing tumor size was observed except for the survival comparison between T3-Add (50-70 mm) and T3-Add (> 70 mm) patients (*P* = 0.330, **Figure S1B**).

### LASSO-penalized multivariable Cox analysis

After PSM, regarding the T3-Add & T2a matched pair, 14 variables were entered into the LASSO models (OS: **Figure S2A**; CSS: **Figure S2C**). The results showed that age, sex, grade, positive lymph nodes, T category, N category and VPI were potential prognostic factors for OS (**Figure S2B**) and grade, positive lymph nodes, T category and N category were potential prognostic factors for CSS (**Figure S2D**). The LASSO-selected variables were further included in the multivariable Cox analysis. The multivariable Cox analysis further confirmed that age (*P* < 0.001), sex (*P* < 0.001), grade (*P* < 0.001), T category (adjusted HR: T3-Add vs. T2a = 1 vs. 0.775, *P* < 0.001), N category (*P* = 0.004) and VPI (*P* = 0.028) were the independent prognostic factors for OS (**Table 2**), and grade (*P* < 0.001), T category (adjusted HR: T3-Add vs. T2a = 1 vs. 0.717, *P* = 0.001) and N category (*P* < 0.001) were the independent prognostic factors for CSS (**Table 2**).

**Table 2:** LASSO-penalized multivariable Cox analysis of the T2a and T3-Add NSCLC patients after PSM.

Variables	OS^a^			CSS^b^		
	HR	95% CI	*P*	HR	95% CI	*P*
Age, years			< 0.001			
Continue	1.033	1.025-1.042				
Sex			< 0.001			
Male	1					
Female	0.656	0.573-0.751				
Grade			< 0.001			< 0.001
Well	1			1		
Moderate	1.605	1.282-2.008		2.223	1.503-3.288	
Poor/undifferentiated	2.261	1.800-2.840		3.587	2.426-5.302	
Positive lymph nodes			0.087			0.118
Continue	1.044	0.994-1.096		1.049	0.988-1.115	
T category			< 0.001			0.001
T3-Add	1			1		
T2a	0.775	0.678-0.886		0.717	0.588-0.875	
N category			0.004			< 0.001
0	1			1		
1	1.340	1.068-1.682		1.762	1.306-2.378	
2	1.632	1.192-2.235		2.171	1.440-3.271	
VPI			0.028			
Without	1					
with	1.179	1.018-1.365				

a Age, sex, grade, positive lymph nodes, T category, N category and VPI were included in the LASSO-penalized Cox model of OS.

b Grade, positive lymph nodes, T category and N category were included in the LASSO-penalized Cox model of CSS.

LASSO, least absolute shrinkage and selection operator; NSCLC, non-small cell lung cancer; PSM, propensity score matching; OS, overall survival; CSS, cancer specific survival; T, tumor; T3-Add, T3 tumors with additional nodules in the same lobe; N, node; VPI, visceral pleural invasion.

Considering the T3-Add & T2b matched pair, the LASSO models selected 5 variables including age, sex, grade, positive lymph nodes and N category for OS, and 2 variables including grade and T category for CSS. In further analyses, the Cox models confirmed that age (*P* < 0.001), sex (*P* < 0.001), grade (*P* < 0.001) and N category (*P* = 0.001) were the independent prognostic factors for OS (**Table 3**), and grade (*P* < 0.001) and N category (*P* < 0.001) were the independent prognostic factors for CSS (**Table 3**).

**Table 3:** LASSO-penalized multivariable Cox analysis of the T2b and T3-Add NSCLC patients after PSM.

Variables	OS^a^			CSS^b^		
	HR	95% CI	*P*	HR	95% CI	*P*
Age, years			< 0.001			
Continue	1.037	1.025-1.048				
Sex			< 0.001			
Male	1					
Female	0.581	0.489-0.689				
Grade			< 0.001			< 0.001
Well	1			1		
Moderate	1.882	1.372-2.581		3.142	1.723-5.728	
Poor/undifferentiated	2.957	2.159-4.048		5.487	3.024-9.955	
Positive lymph nodes			0.082			
Continue	1.047	0.994-1.103				
N category			0.001			< 0.001
0	1			1		
1	1.322	1.006-1.737		1.864	1.329-2.616	
2	1.997	1.376-2.900		3.301	2.261-4.821	

a Age, sex, grade, positive lymph nodes and N category were included in the LASSO-penalized Cox model of OS.

b Grade and T category were included in the LASSO-penalized Cox model of CSS.

LASSO, least absolute shrinkage and selection operator; NSCLC, non-small cell lung cancer; PSM, propensity score matching; T3-Add, T3 tumors with additional nodules in the same lobe; OS, overall survival; CSS, cancer specific survival; N, node.

## Discussion

The current study evaluated the prognosis of the T3-Add NSCLC patients from a large public database. Our results suggested that the survivals of the T3-Add patients were superior to those of the T3 patients, but were comparable to those of the T2b patients after balancing the baseline characteristics. The LASSO-penalized Cox models further confirmed that the T category (T3-Add vs. T2b) was not a prognostic factor for the patients in the T3-Add & T2b matched pair. Therefore, we proposed that it is necessary to reconsider the T category of the patients with additional nodules in the same lobe in the forthcoming 9^th^ edition of TNM staging manual. This gave us a hint, but more validations are still warranted.

In this study, the incidence of the T3-Add patients was 5.6% (2,312/41,370). The 5-year OS rate of the T3-Add patients was 51.5%, which was higher than the previous studies, where the authors reported the rate of 42.0% (8, 9). A possible explanation for this difference might be that a portion of T3-Add patients had not received surgery in their cohorts, whereas all included patients had undergone surgical resection in this study. To date, surgery is still the preferred treatment for these patients (12). We reported for the first time that the 5-year CSS rate of the T3-Add patients was 72.5%, which was better than previously thought.

In the current TNM staging manual, T3 category descriptors include tumor size greater than 5 cm but less than or equal to 7 cm, tumors with additional nodules in the same lobe and tumors with parietal pleural, chest wall, pericardium or phrenic nerve invasion (4). In the analysis of the classification of lung cancer with separate tumor nodules, the results of the IASLC lung cancer staging project showed a trend toward longer OS in the T3-Add group when compared with other T3 groups, but the result was not statistically significant (9). Therefore, the project proposed that tumors with same-lobe tumor nodules should be classified as T3. However, controversy exists on the T category of these patients. A Netherland study analyzed the data of 683 pT3N0M0 NSCLC patients and demonstrated that the adenocarcinoma subgroup of the T3-Add patients and T2 patients had comparable survival rates, whereas the T3-Add patients in the squamous cell carcinoma subgroup should remain classified as T3 category (13).

To date, only one study had externally validated the current T2b and T3-Add category developed by IASLC (4). In the study by Kumar et al. (8), the authors reviewed 31,563 T2b-3N0-3M0 NSCLC patients and demonstrated that the T2b and T3-Add patients had similar 5-year OS rates (53.4% vs. 52.3%). So, they concluded that this finding should be taken into consideration in the forthcoming 9^th^ edition of TNM staging manual. The strengths of their study were the large number of cases in several subgroups and the implementation of PSM method. However, clinical TNM stage was used in their study which could lead to bias. In addition, only OS was evaluated, which might limit clinical reference value. In our study, only resected patients were included to ensure the accuracy of staging. We also analyzed the CSS differences between these two groups of patients. It is known that the older patients have a high rate of comorbidities, which could lead to a high risk of competing non-cancer related events. In this study, the median age of included patients was 69 years. In addition, the 5-year CSS rate of the T3-Add patients was much better than the corresponding 5-year OS rate (72.5% vs. 51.5%). Therefore, it is necessary to explore the CSS differences between these two groups.

Our study is meaningful. Our study revealed that the survivals of the T3-Add patients was comparable to those of the T2b patients, which added to the evidence on the topic that it is necessary to reconsider the T category for the NSCLC patients with additional nodules in the same lobe. The accurate TNM staging is crucial to estimate patients’ prognosis and drive subsequent treatments selection. Although the current National Comprehensive Cancer Network (NCCN) guideline (12) for NSCLC recommends nondifferential treatment modalities for the T3-Add and T2b patients, in the real-world setting, the former one is less likely to receive surgery than the latter one. It is reported that only half of the T3-Add patients were treated surgically as compared with up to 90% of the T2b patients (8). The reason behind the difference was that surgery was not included in the initial treatments for most of the T3-Add patient with non-operative management because the physicians did not recommend it (8). In our view, just like T2b patients, T3-Add patients do benefit from surgery, and these patients should be treated properly.

Our study had some limitations. First, the information about additional tumor nodules recorded in the SEER data set is not specific, which only includes a simple description that CS Site-Specific Factor 1 code 010: separate tumor nodules in the same lobe of ipsilateral lung. The rigor definition of T3-Add is that a solid lung tumor with at least one additional tumor nodule of similar imaging appearance and matching histologic appearance (14), and the information is not recorded in the SEER data set. However, due to the low incidence of the T3-Add tumors, it might be hard to draw a conclusion with strong statistical power from a single institute. Therefore, the superiority of large number of cases deposited in the SEER data set is reflected. Second, the common drawback of the public data set is the rough deposited data. In this study, other important prognostic variables for example comorbidity, surgical margin and genetic and molecular factors are lacking. Further efforts on multicenter study results collection and broader clinicopathological variables recruitment are encouraged. At last, although PSM method was used in this study, the retrospective design may have contributed to bias. We hoped future works could validate our results.

## Conclusion

T3-Add and T2b NSCLC patients had similar survivals, and we proposed that it is necessary to reconsider the T category for the NSCLC patients with additional nodules in the same lobe in the forthcoming 9^th^ edition of TNM staging manual.

## Data availability statement

The datasets presented in this study can be found in online repositories. The names of the repository/repositories and accession number(s) can be found below: SEER database (https://seer.cancer.gov/).

## Ethics statement

The studies involving human participants were reviewed and approved by Peking University People’s Hospital. Written informed consent for participation was not required for this study in accordance with the national legislation and the institutional requirements.

## Author contributions

XW and FY: Conceptualization, Supervision, Writing-Reviewing and Editing. J-SC: Methodology, Software, Data curation, Writing-Original draft preparation. XW: Data curation. All authors contributed to the article and approved the submitted version.

## Supplementary material

Supplementary Figure 1 Kaplan–Meier estimates of survivals in T3-Add patients stratified by tumor size. **(A)** overall survival: T3-Add (0-30 mm) vs. T3-Add (30-50 mm) vs. T3-Add (50-70 mm) vs. T3-Add (> 50 mm); **(B)** cancer specific survival: T3-Add (0-30 mm) vs. T3-Add (30-50 mm) vs. T3-Add (50-70 mm) vs. T3-Add (> 50 mm). T3-Add, T3 non-small cell lung cancer with additional tumor nodules in the same lobe.

Supplementary Figure 2 Prognostic variables selection using the LASSO regression model in the T3-Add & T2a pair after PSM. LASSO coefficient profiles of 14 variables against the log (Lambda) sequence for overall survival **(A)** and cancer specific survival **(C)**. Tuning parameter (Lambda) selection in the LASSO model used 10-fold cross-validation *via* minimum criteria (overall survival: **(B)** cancer specific survival: **(D)**. LASSO, least absolute shrinkage and selection operator; PSM, propensity score matching.

Supplementary Figure 3 Prognostic variables selection using the LASSO regression model in the T3-Add & T2b pair after PSM. LASSO coefficient profiles of 14 variables against the log (Lambda) sequence for overall survival **(A)** and cancer specific survival **(C)**. Tuning parameter (Lambda) selection in the LASSO model used 10-fold cross-validation *via* minimum criteria (overall survival: **(B)** cancer specific survival: **(D)**. LASSO, least absolute shrinkage and selection operator; PSM, propensity score matching.
