# Supplementary figures and images for: Reconsidering the T category for the T3 non-small cell lung cancer with additional tumor nodules in the same lobe: A population-based study
Source: Front Oncol. 2023 Apr 5;13:1043386. doi: 10.3389/fonc.2023.1043386 (PMC10113646; doi:10.3389/fonc.2023.1043386)

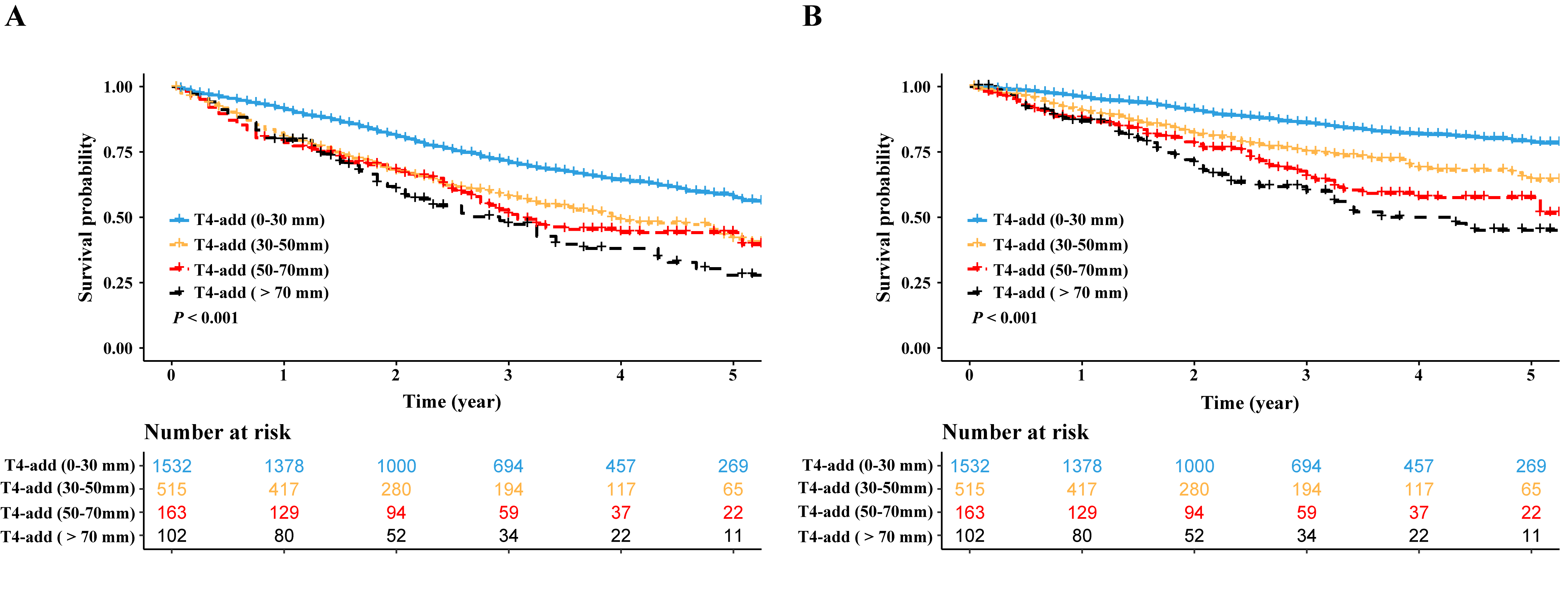

Supplement: Supplementary Figure 1 — Kaplan–Meier estimates of survivals in T3-Add patients stratified by tumor size. (A) overall survival: T3-Add (0-30 mm) vs. T3-Add (30-50 mm) vs. T3-Add (50-70 mm) vs. T3-Add (> 50 mm); (B) cancer specific survival: T3-Add (0-30 mm) vs. T3-Add (30-50 mm) vs. T3-Add (50-70 mm) vs. T3-Add (> 50 mm). T3-Add, T3 non-small cell lung cancer with additional tumor nodules in the same lobe. [file Image_1.tif]

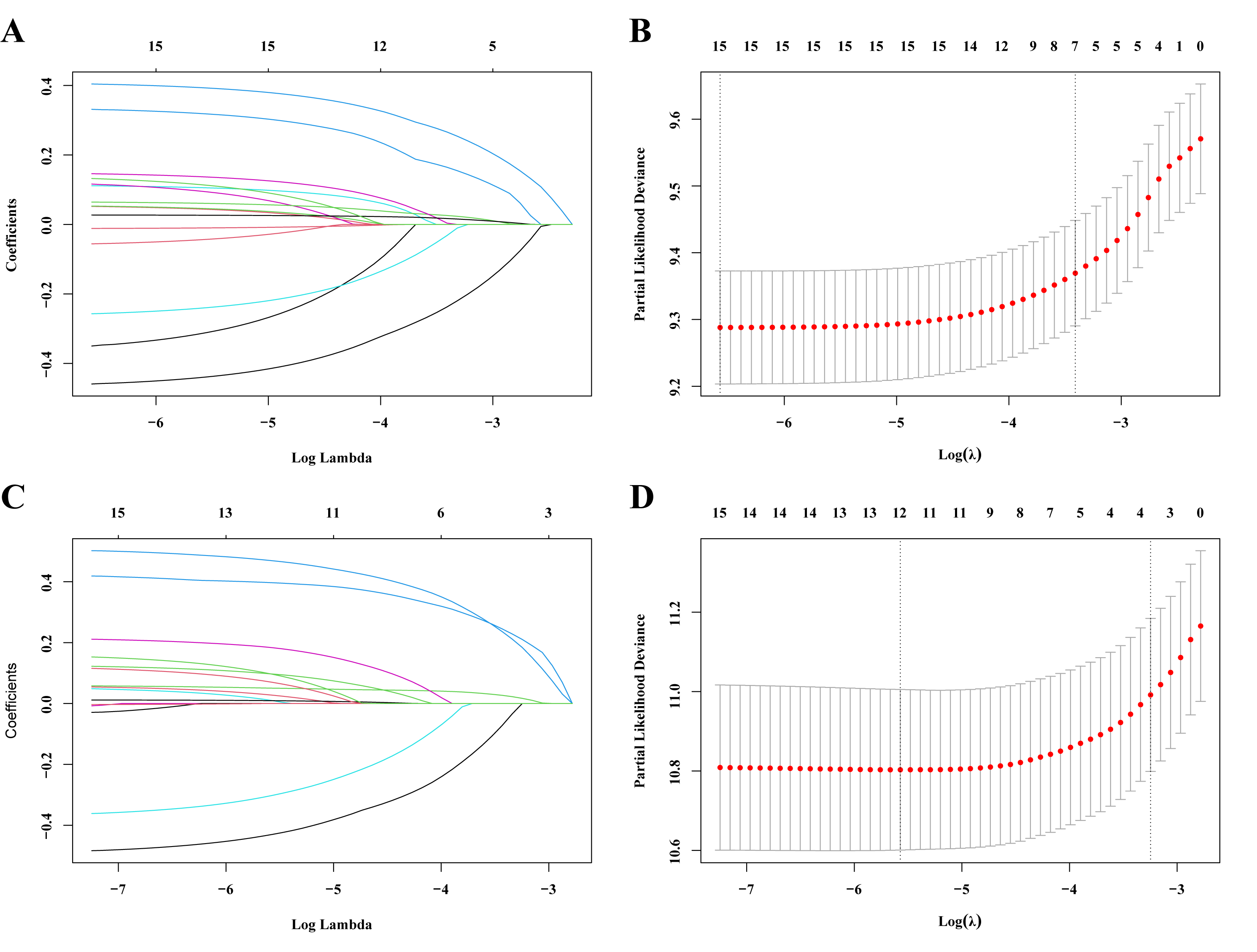

Supplement: Supplementary Figure 2 — Prognostic variables selection using the LASSO regression model in the T3-Add & T2a pair after PSM. LASSO coefficient profiles of 14 variables against the log (Lambda) sequence for overall survival (A) and cancer specific survival (C). Tuning parameter (Lambda) selection in the LASSO model used 10-fold cross-validation via minimum criteria (overall survival: (B) cancer specific survival: (D). LASSO, least absolute shrinkage and selection operator; PSM, propensity score matching. [file Image_2.tif]

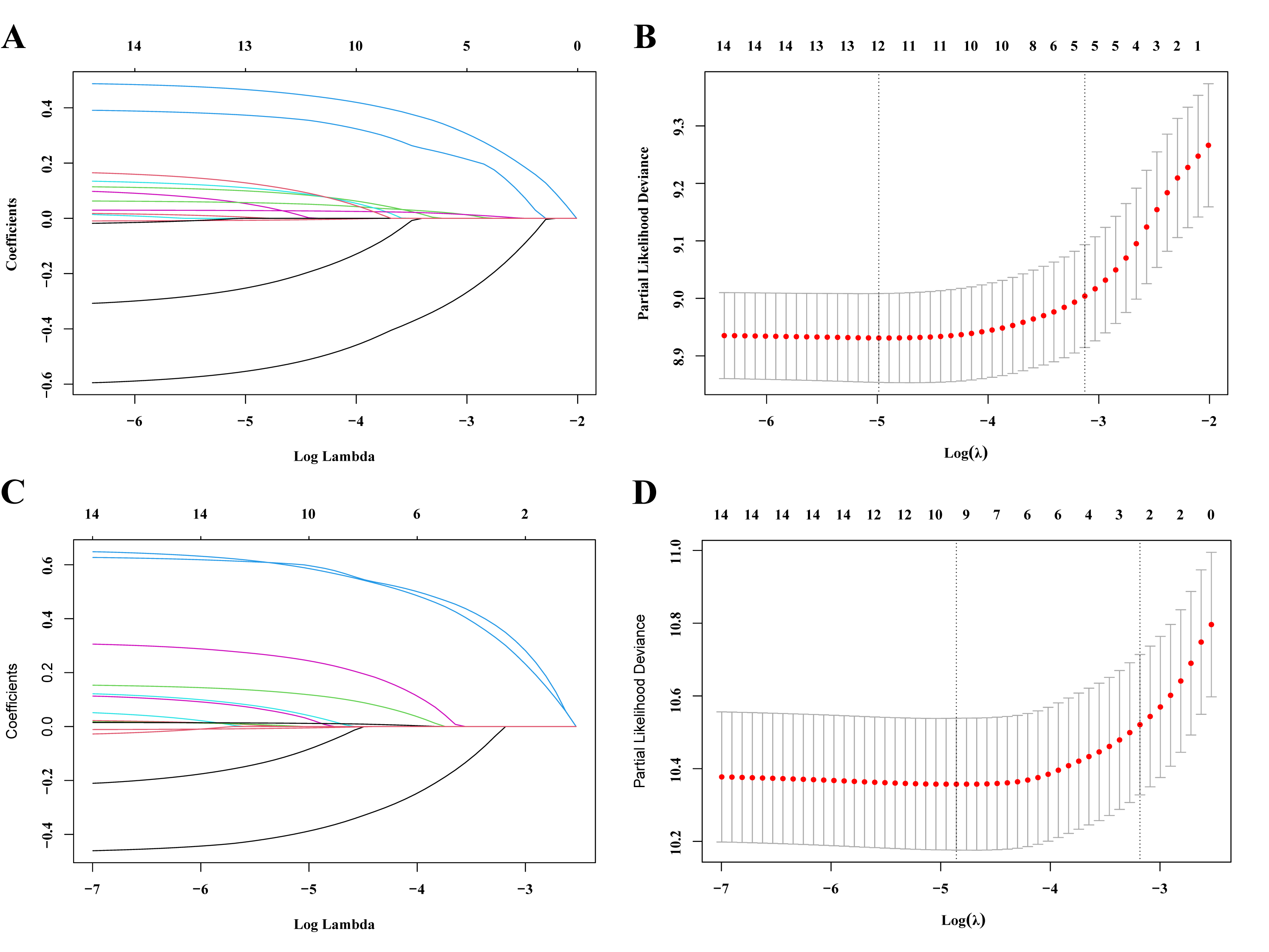

Supplement: Supplementary Figure 3 — Prognostic variables selection using the LASSO regression model in the T3-Add & T2b pair after PSM. LASSO coefficient profiles of 14 variables against the log (Lambda) sequence for overall survival (A) and cancer specific survival (C). Tuning parameter (Lambda) selection in the LASSO model used 10-fold cross-validation via minimum criteria (overall survival: (B) cancer specific survival: (D). LASSO, least absolute shrinkage and selection operator; PSM, propensity score matching. [file Image_3.tif]
