# Supplementary material for: Reconsidering the T category for the T3 non-small cell lung cancer with additional tumor nodules in the same lobe: A population-based study
Source: Front Oncol. 2023 Apr 5;13:1043386. doi: 10.3389/fonc.2023.1043386 (PMC10113646; doi:10.3389/fonc.2023.1043386)
Supplement: Supplementary file 4 [file Table_1.docx]

**Table S1. The baseline characteristics of the T3-Add & T2 pair and the T3-Add & T3 pair after PSM**

| Variables | T3-Add (N=1,623) | T2 (N=1,623) | *P* | T3-Add (N=650) | T3 (N=650) | *P* |
| --- | --- | --- | --- | --- | --- | --- |
| Age, years |  |  | 0.197^a^ |  |  | 0.849^a^ |
| Median (range) | 69 (37-88) | 70 (30-91) |  | 70 (49-89) | 70 (49-89) |  |
| Sex |  |  | 0.549 |  |  | 0.868 |
| Male | 737 (45.4) | 754 (46.5) |  | 333 (51.2) | 336 (51.7) |  |
| Female | 886 (54.6) | 869 (53.5) |  | 317 (48.8) | 314 (48.3) |  |
| Histology |  |  | 0.358 |  |  | 0.967 |
| Adenocarcinoma | 946 (58.3) | 981 (60.4) |  | 346 (53.2) | 342 (52.6) |  |
| Squamous cell carcinoma | 296 (18.2) | 293 (18.1) |  | 181 (27.8) | 185 (28.5) |  |
| Other | 381 (23.5) | 349 (21.5) |  | 123 (18.9) | 123 (18.9) |  |
| Grade |  |  | 0.573 |  |  | 0.985 |
| Well | 306 (18.9) | 296 (18.2) |  | 69 (10.6) | 69 (10.6) |  |
| Moderate | 738 (45.5) | 768 (47.3) |  | 288 (44.3) | 291 (44.8) |  |
| Poor/undifferentiated | 579 (35.7) | 559 (34.4) |  | 293 (45.1) | 290 (44.6) |  |
| Surgery |  |  | 0.307 |  |  | 1.000^b^ |
| Lobectomy | 1,504 (92.7) | 1,481 (91.3) |  | 636 (97.8) | 636 (97.8) |  |
| Pneumonectomy | 26 (1.6) | 34 (2.1) |  | 3 (0.5) | 3 (0.5) |  |
| Sublobectomy | 93 (5.7) | 108 (6.7) |  | 11 (1.7) | 11 (1.7) |  |
| Radiotherapy |  |  | 0.135 |  |  | 0.759 |
| No | 1,507 (92.9) | 1,528 (94.1) |  | 629 (96.8) | 627 (96.5) |  |
| Yes | 116 (7.1) | 95 (5.9) |  | 21 (3.2) | 23 (3.5) |  |
| Chemotherapy |  |  | 0.938 |  |  | 0.904 |
| No | 1,159 (71.4) | 1,161 (71.5) |  | 449 (69.1) | 451 (69.4) |  |
| Yes | 464 (28.6) | 462 (28.5) |  | 201 (30.9) | 199 (30.6) |  |
| N category |  |  | 0.838 |  |  | 0.988 |
| 0 | 1,266 (78.0) | 1,279 (78.8) |  | 558 (85.8) | 558 (85.8) |  |
| 1 | 212 (13.1) | 207 (12.8) |  | 58 (8.9) | 59 (9.1) |  |
| 2 | 145 (8.9) | 137 (8.4) |  | 34 (5.2) | 33 (5.1) |  |
| VPI |  |  | 0.720 |  |  | 1.000 |
| Without | 1,199 (73.9) | 1,190 (73.3) |  | 515 (79.2) | 515 (79.2) |  |
| With | 424 (26.1) | 433 (26.7) |  | 135 (20.8) | 135 (20.8) |  |

a Mann–Whitney U test

b Fisher’s exact test

T, tumor; T3-Add, T3 tumors with additional nodules in the same lobe; PSM, propensity score matching; N, node; VPI, visceral pleural invasion.

**Table S2. The baseline characteristics of the T3-Add & T2a pair and the T3-Add & T2b pair after PSM**

| Variables | T3-Add (N=1,381) | T2a (N=1,381) | *P* | T3-Add (N=788) | T2b (N=788) | *P* |
| --- | --- | --- | --- | --- | --- | --- |
| Age, years |  |  | 0.801^a^ |  |  | 0.940^a^ |
| Median (range) | 70 (32-88) | 69 (25-96) |  | 70 (44-88) | 70 (35-88) |  |
| Sex |  |  | 0.378 |  |  | 0.920 |
| Male | 598 (43.3) | 621 (45.0) |  | 401 (50.9) | 399 (50.6) |  |
| Female | 783 (56.7) | 760 (55.0) |  | 387 (49.1) | 389 (49.4) |  |
| Histology |  |  | 0.835 |  |  | 0.969 |
| Adenocarcinoma | 827 (59.9) | 812 (58.8) |  | 456 (57.9) | 454 (57.6) |  |
| Squamous cell carcinoma | 238 (17.2) | 247 (17.9) |  | 176 (22.3) | 180 (22.8) |  |
| Other | 316 (22.9) | 322 (23.3) |  | 156 (19.8) | 154 (19.5) |  |
| Grade |  |  | 0.941 |  |  | 0.997 |
| Well | 271 (19.6) | 266 (19.3) |  | 130 (16.5) | 129 (16.4) |  |
| Moderate | 649 (47.0) | 646 (46.8) |  | 359 (45.6) | 360 (45.7) |  |
| Poor/undifferentiated | 461 (33.4) | 469 (34.0) |  | 299 (37.9) | 299 (37.9) |  |
| Surgery |  |  | 0.375 |  |  | 0.905^b^ |
| Lobectomy | 1,284 (93.0) | 1,265 (91.6) |  | 773 (98.1) | 772 (98.0) |  |
| Pneumonectomy | 19 (1.4) | 25 (1.8) |  | 2 (0.3) | 3 (0.4) |  |
| Sublobectomy | 78 (5.6) | 91 (6.6) |  | 13 (1.6) | 13 (1.6) |  |
| Radiotherapy |  |  | 0.863 |  |  | 0.681 |
| No | 1,309 (94.8) | 1,311 (94.9) |  | 759 (96.3) | 762 (96.7) |  |
| Yes | 72 (5.2) | 70 (5.1) |  | 29 (3.7) | 26 (3.3) |  |
| Chemotherapy |  |  | 0.619 |  |  | 0.860 |
| No | 1,068 (77.3) | 1,057 (76.5) |  | 596 (75.6) | 599 (76.0) |  |
| Yes | 313 (22.7) | 324 (23.5) |  | 192 (24.4) | 189 (24.0) |  |
| N category |  |  | 0.832 |  |  | 0.945 |
| 0 | 1,142 (82.7) | 1,137 (82.3) |  | 667 (84.6) | 670 (85.0) |  |
| 1 | 146 (10.6) | 155 (11.2) |  | 77 (9.8) | 77 (9.8) |  |
| 2 | 93 (6.7) | 89 (6.4) |  | 44 (5.6) | 41 (5.2) |  |
| VPI |  |  | 0.931 |  |  | 0.786 |
| Without | 1,023 (74.1) | 1,021 (73.9) |  | 656 (83.2) | 660 (83.8) |  |
| With | 358 (25.9) | 360 (26.1) |  | 132 (16.8) | 128 (16.2) |  |

a Mann–Whitney U test

b Fisher’s exact test

T, tumor; T3-Add, T3 tumors with additional nodules in the same lobe; PSM, propensity score matching; N, node; VPI, visceral pleural invasion.
